# Comparing the Effect of Multiple Histone Deacetylase Inhibitors on SSTR2 Expression and [^111^In]In-DOTATATE Uptake in NET Cells

**DOI:** 10.3390/cancers13194905

**Published:** 2021-09-29

**Authors:** Maria J. Klomp, Simone U. Dalm, Peter M. van Koetsveld, Fadime Dogan, Marion de Jong, Leo J. Hofland

**Affiliations:** 1 Department of Radiology and Nuclear Medicine, Erasmus MC, 3015 GD Rotterdam, The Netherlands; m.j.klomp@erasmusmc.nl (M.J.K.); s.dalm@erasmusmc.nl (S.U.D.); 2 Department of Internal Medicine, Division of Endocrinology, Erasmus MC, 3015 GD Rotterdam, The Netherlands; p.vankoetsveld@erasmusmc.nl (P.M.v.K.); f.dogan@erasmusmc.nl (F.D.)

**Keywords:** neuroendocrine tumors, peptide receptor radionuclide therapy, somatostatin type-2 receptors, SSTR2, [^111^In]In-DOTATATE, upregulation, epigenetic drugs, histone deacetylase inhibitors

## Abstract

**Simple Summary:**

Patients diagnosed with neuroendocrine tumors (NETs) are often treated with peptide receptor radionuclide therapy (PRRT). This therapy targets the somatostatin type-2 receptors (SSTR2) frequently overexpressed on these types of tumors. Although this therapy has proven to be effective, complete responses are rare and therapy improvement is desirable. We aimed to increase SSTR2 expression on NET cells, potentially increasing the number of patients eligible for SSTR2-targeted PRRT and improving clinical outcomes. We used histone deacetylase inhibitors (HDACis) to manipulate the epigenetic machinery and hereby aimed to increase SSTR2 gene transcription. Our results showed that the HDACis increased SSTR2 expression in several NET cell lines. Moreover, the uptake of radiolabeled DOTATATE, the tracer used for PRRT, was enhanced. The observed reversibility profile after HDACi withdrawal of the induced effects suggests that proper timing of HDACi treatment is likely essential.

**Abstract:**

The aim of this study was to increase somatostatin type-2 receptor (SSTR2) expression on neuroendocrine tumor (NET) cells using histone deacetylase inhibitors (HDACis), potentially increasing the uptake of SSTR2-targeted radiopharmaceuticals and subsequently improving treatment efficacy of peptide receptor radionuclide therapy (PRRT). Human NET cell lines BON-1, NCI-H727, and GOT1 were treated with HDACis (i.e., CI-994, entinostat, LMK-235, mocetinostat, panobinostat, or valproic acid (VPA); entinostat and VPA were the HDACis tested in GOT1 cells) to examine *SSTR2* mRNA expression levels and uptake of SSTR2-targeting radiotracer [^111^In]In-DOTATATE. Reversibility of the induced effects was examined after drug-withdrawal. Finally, the effect of VPA on radiosensitivity was investigated. A strong stimulatory effect in BON-1, NCI-H727, and GOT1 cells was observed after HDACi treatment, both on *SSTR2* mRNA expression levels and [^111^In]In-DOTATATE uptake. The effects of the HDACis were largely reversible over a period of seven days, demonstrating largest reductions within the first day. The reversibility profile of the induced effects suggests that proper timing of HDACi treatment is most likely essential for a beneficial outcome. In addition to increasing SSTR2 expression levels, VPA enhanced the radiosensitivity of all cell lines. In conclusion, HDACi treatment increased SSTR2 expression, and radiosensitivity was also enhanced upon VPA treatment.

## 1. Introduction

Neuroendocrine tumors (NETs) form a heterogeneous group of tumors which are often metastasized upon time of diagnosis. Unfortunately, treatment options for NETs are still limited [1]. The frequent overexpression of the somatostatin type-2 receptor (SSTR2) forms a pivotal target for therapy. Treatment with somatostatin analogues (SSAs) and the subsequent development of radiolabeled SSAs, i.e., [^177^Lu]Lu-[DOTA-Tyr3]octreotate ([^177^Lu]Lu-DOTATATE) used for peptide receptor radionuclide therapy (PRRT), have both proven their efficacy in the treatment of NETs [2,3,4]. Unfortunately, complete responses after PRRT are still rare [4,5]. Several promising approaches are under investigation to improve the efficacy of PRRT, such as SSTR2 upregulation using epigenetic drugs.

Varying SSTR2 expression levels among patients [6] and the absence of known mutations in the human SSTR2 gene, indicate that its expression may be regulated by epigenetic mechanisms, rather than genetic mutations. Previous studies have described an important role for epigenetic regulation in both NET pathogenesis and SSTR2 expression [7,8,9,10]. Due to the prominent role of epigenetics in this disease, it is hypothesized that epigenetic drugs will mainly target the tumor cells and, to a lesser extent, control tissue. By using synthetic inhibitors targeting the epigenetic machinery, it is possible to modify the epigenetic landscape. For the scope of this study, we focused only on histone acetylation. Histone deacetylase inhibitors (HDACis) specifically target histone deacetylases. Inhibition of these enzymes results in stimulation of the active euchromatin state, the state in which DNA is actively being transcribed. In short, the use of HDACis may modify the epigenetic profile in such a way that protein expression levels are increased [11,12].

In this study, the aim was to thoroughly compare the effect of different HDACis targeting several classes of HDAC enzymes in three NET models: BON-1, NCI-H727, and GOT1 cells derived from pancreatic NET, lung carcinoid tumor, and midgut NET, respectively. We evaluated the effect of the selected HDACis on the different NET cell lines with respect to *SSTR2* mRNA expression levels and SSTR2 functionality using [^111^In]In-DOTATATE uptake studies. Moreover, we analyzed reversibility profiles over time after HDACi withdrawal and investigated the radiosensitivity upon exposure to one of the HDACis.

## 2. Materials and Methods

### 2.1. Cell Culture

The human pancreatic neuroendocrine tumor cell line BON-1 (kind gift of Dr. Townsend, University of Texas Medical branch, Galveston, TX, USA), the human pulmonary carcinoid cell line NCI-H727 (ATCC CRL-5815), and the human midgut neuroendocrine tumor cell line GOT1 (kind gift of Ola Nilsson, Sahlgrenska Cancer Center, University of Gothenburg, Sweden) were used in this study. BON-1 cells were cultured in DMEM/F-12 (1:1) supplemented with 10% (*v*/*v*) FCS, 2 mM L-glutamine, 1.25 mg/L fungizone, and 1 × 10^5^ U/L penicillin; NCI-H727 cells were cultured in RPMI medium 1640 + L-glutamine supplemented with 10% (*v*/*v*) FCS, 100 U/mL penicillin, and 100 µg/mL streptomycin, and; GOT-1 cells were cultured in RPMI medium 1640 supplemented with 10% (*v*/*v*) FCS, 2 mM L-glutamine, 100 U/mL penicillin, 100 µg/mL streptomycin, 1.0 g/L insulin, 0.55 g/L transferrin, and 67 µg/L selenite. Once a week, BON-1 and NCI-H727 cells were trypsinized using 0.05% (*v*/*v*) trypsin + 0.53 mM EDTA and fresh medium was added on day four. GOT1 cells were trypsinized every two weeks using 0.05% (*v*/*v*) trypsin + 0.53 mM EDTA supplemented with DNAse (2 U/mL) with medium refreshment after one week.

### 2.2. Histone Deacetylase Inhibitors

Six HDACis were tested in this study: valproic acid sodium salt (VPA; Sigma-Aldrich, Zwijndrecht, The Netherlands), entinostat (ENT; Sigma-Aldrich), CI-994 (Sigma-Aldrich), LMK-235 (AbMole Bioscience Inc., Brussels, Belgium), mocetinostat (MOC; Selleck Chemicals LCC, Breda, The Netherlands), and panobinostat (PAN; Selleck Chemicals LCC, Breda, The Netherlands). All HDACis were dissolved in 40% DMSO, except for VPA, which was dissolved in sterile aquadest. In all experiments, a final concentration of 0.4% DMSO or 1.0% aquadest was reached in the culture medium of treatment groups and vehicle controls. The tested HDACis were targeting HDACs, which are divided into several classes; class I (HDAC1, HDAC2, HDAC3, HDAC8), class IIA (HDAC4, HDAC5, HDAC7, HDAC9), class IIB (HDAC6, HDAC10), and class IV (HDAC11) [13]. PAN targeted class I, IIA, IIB; MOC targeted HDAC1, HDAC2, and HDAC3 in class I and HDAC11 in class IV; ENT, VPA, and CI-994 targeted HDAC1, HDAC2, and HDAC3 in class I and LMK-235 targeted HDAC4 and HDAC5 in class IIA [14,15].

### 2.3. Dose–Response Curves and DNA Quantification

For all HDACis, dose–response studies based on a seven-day treatment schedule were performed. One day before HDACi treatment started, cells were plated in 24-well plates. Drug-supplemented medium was refreshed on day three and medium was removed on day seven. A cell growth assay was then performed by DNA quantification using Hoechst 33256 as previously described [16] with the adjustment of using 0.2% (*v*/*v*) Triton X-100 for cell lysis. For GOT1 cells, Quant-iT PicoGreen dsDNA reagent (Invitrogen, Breda, The Netherlands) was used. For this, Quant-iT PicoGreen dsDNA was diluted 120 times and 20 µL was added to each well. The absorbance was subsequently measured at excitation and emission wavelengths of 485 nm and 535 nm, respectively.

### 2.4. HDACi-Treatment Regimen

To study the effect of HDACis on SSTR2-expression, cells were plated in T75 flasks on day zero. HDACis were added on day one at their IC_50_ growth inhibitory concentrations and drug-supplemented medium was refreshed on day three. On day five, cells were trypsinized and plated for further analysis. Exactly four hours after cell plating, HDACis were added again. On day seven, samples were collected for analysis by RT-qPCR analysis (24-well plates) and for internalization studies (12-well plates). For reversibility and radiosensitivity studies (24-well plates), culture periods were prolonged for another week in the absence and presence of HDACis, respectively.

### 2.5. mRNA Analysis

For mRNA analysis, cells were lysed and subsequently incubated with oligo(dT)_25_ dynabeads (Invitrogen, Breda, The Netherlands) to isolate poly-A^+^ mRNA. Then, 23 µL H_2_O was added for elution, and 10 µL poly-A^+^ mRNA was used in the next steps. Poly-A^+^ mRNA was converted into cDNA using the commercial RevertAid First Strand cDNA synthesis kit (Thermo Scientific, Breda, The Netherlands). To exclude the possibility of DNA contamination, cDNA was also prepared without the addition of RevertAid Reverse Transcriptase. Samples were diluted by adding 180 µL H_2_O and RT-qPCR was performed. In short, 5 µL sample was mixed with 7.5 µL Taqman Universal PCR mastermix (Applied Biosystems, Breda, The Netherlands) supplemented with primers and probes. SSTR2 expression was determined relative to three housekeeping genes (HKGs). For analysis, the QuantStudio 7 Flex RT-qPCR system with QuantStudio Real-Time PCR software v1.5 was used. The number of copies for SSTR2 and all HKGs was calculated by the efficiency factor to the power of ∆Ct (i.e., 40 minus measured Ct). Subsequently, the relative SSTR2 expression was calculated by dividing the number of SSTR2 copies by the geometric mean of all HKGs. Details on primers are represented in Table S1.

### 2.6. [^111^In]In-DOTATATE Radiolabeling

DOTATATE (Bachem AG, Budendorf, Switzerland) was radiolabeled with [^111^In]InCl_3_ (Curium Pharma, Petten, The Netherlands) with a molar activity of 50 MBq/nmol as previously described [17]. The radiochemical yield and radiochemical purity, measured using thin-layer chromatography and high-performance liquid chromatography, respectively, as previously described [17], were >95% and RCP > 90%, respectively.

### 2.7. [^111^In]In-DOTATATE Internalization Studies

Internalization studies were performed as previously described [18]. Cells were incubated with internalization medium (DMEM (1x)–GlutaMAX-I, 1% (wt/v) BSA, and 20 mM HEPES (pH 7.4)) supplemented with 10^−9^ M [^111^In]In-DOTATATE (50 MBq/1 nmol), with or without 10^−6^ M unlabeled DOTATATE, for four hours. Following incubation, the excess unbound radiotracer was removed and the membrane-bound and internalized radioactivity were determined. For GOT1 cells, the protocol was slightly adjusted due to insufficient cell adherence. In each step, non-adherent cells were pelleted by centrifugation (3.5× *g*, 5 min) and combined with the attached cells. In addition, the total uptake was determined for GOT1 cells. To correct for possible differences in cell numbers, cell pellets of additional wells were collected and DNA content was measured with Hoechst 33258 using the same protocol described previously.

### 2.8. Reversibility

To determine the reversibility of HDACi-induced effects, drug-supplemented medium was removed on day seven. Cells were subsequently maintained in normal growth medium up to an additional period of seven days, with medium renewal after three days. Samples were collected one, three, and seven days after drug withdrawal and analyzed by RT-qPCR using the previously described method.

### 2.9. Radiosensitivity

HDACi-supplemented growth medium was refreshed on day seven and cells were irradiated up to 8 grays (Gy) using the RS320 (Xstrahl Live Sciences; 1.6554 Gy/min, 195 kV, 10 mA). On day eleven, HDACi-supplemented growth medium was refreshed again. Cells were fixated on day 14 using 10% (wt/v) trichloroacetic acid. Subsequently, plates were incubated with 0.5% (wt/v) sulforodamine B (SRB) solution. After incubation, the excess SRB solution was removed by washing plates with 1% (*v*/*v*) acetic acid and protein-bound dye was solubilized with 10 mM tris-base solution. Using a SpectraMax iD3 plate reader (Molecular Devices), the optical density was measured at 560 nm. For each plate, a background measurement was included.

### 2.10. Statistical Analysis

To estimate IC_50_ values, the obtained percentages were plotted using spline/LOWESS analysis using the point-to-point curve. For detecting correlations, the Pearson r^2^ was determined. For all other analysis, results were calculated as percentage increase or decrease compared to the control situation. The resulting percentages were log-transformed. One-way ANOVA analysis using the Tukey post-hoc test was performed to detect significant differences between HDACi-treated cells. To detect differences in radiosensitivity and obtain IC_50_ values, a dose–response curve was plotted with variable slope. For all experiments, both biological and technical replicates were included. All results represent the mean ± SD of at least two independent biological replicates and at least three technical replicates; * *p* < 0.05, ** *p* < 0.01, *** *p* < 0.001, NS; non-significant. All statistical analyses were performed using GraphPad Prism 5 software.

## 3. Results

### 3.1. NET Cell-Line Characterization

*SSTR2* expression levels were 0.0038 ± 0.0005, 0.0055 ± 0.0015, and 0.1468 ± 0.0248 (corrected for the geometric mean of three HKGs) in BON-1, NCI-H727, and GOT1 cells, respectively. Consistent with this, the internalized fraction of [^111^In]In-DOTATATE was 6.99 ± 1.75 and 40.10 ± 9.78 percentage added dose per milligram DNA (%AD/mg DNA) in BON-1 and NCI-H727 cells, respectively. In line with mRNA expression levels, the total uptake in GOT1 cells was the highest: 405.04 ± 98.12% AD/mg DNA. *SSTR2* mRNA expression levels and uptake of radiolabeled [^111^In]In-DOTATATE significantly correlated with an r^2^ of 0.9958 (*p* = 0.0413) (Figure S1).

### 3.2. Effects of HDACis in BON-1 and NCI-H727 Cells

**Figure 1:**
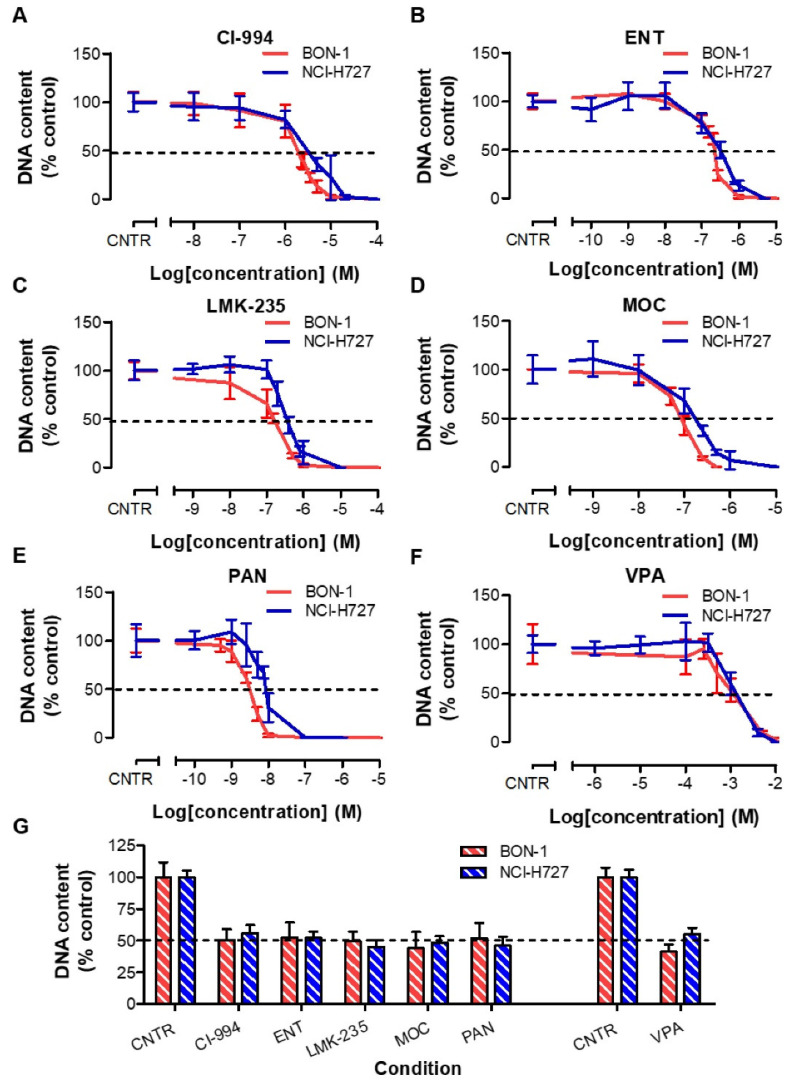
(**A**–**F**) Dose–response curves of six HDACis in BON-1 and NCI-H727 cells, represented in red and blue, respectively. (**G**) In order to include an experiment for further analysis, in which the effects of HDACis on *SSTR2* mRNA levels, uptake of [^111^In]In-DOTATATE and radiosensitivity were evaluated, a proper reduction in DNA amount (i.e., approximately 50%) was confirmed as the internal control for each experiment. As HDACis were either dissolved in 40% DMSO or sterile aquadest, two vehicle-controls were included.

BON-1 cells showed to be slightly more sensitive for HDACi treatment than NCI-H727 cells. The IC_50_ values on growth of BON-1 and NCI-H727 cells were, respectively, 1.85 µM and 3.05 µM for CI-994, 218 nM and 315 nM for ENT, 154 nM and 348 nM for LMK-235, 84.3 nM and 171 nM for MOC, 3.11 nM and 8.53 nM for PAN, and 1.12 mM and 1.31 mM for VPA (Figure 1A–F, Table S2). Treatment at these IC_50_ values demonstrated an expected inhibition of approximately 50% for all HDACis (Figure 1G).

**Figure 2:**
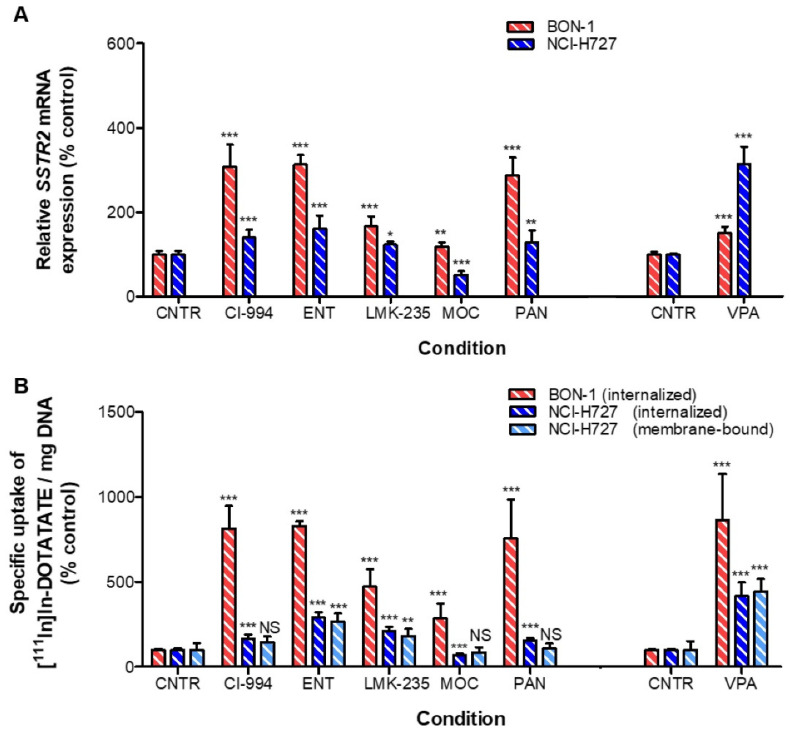
NET cells lines were treated for seven days with HDACis at the IC_50_ dose, and subsequently analyzed by (**A**) RT-qPCR (*SSTR2* mRNA) and (**B**) [^111^In]In-DOTATATE uptake studies. For uptake studies, DNA was quantified to correct for differences in cell number at the start of the assay. Membrane-bound fractions for BON-1 are not shown as values were too low for accurate measurement. Results were normalized to vehicle-treated cells. * *p* < 0.05, ** *p* < 0.01, *** *p* < 0.001, NS; non-significant.

In BON-1 cells, all HDACis significantly increased *SSTR2* mRNA expression levels (*p* < 0.001). CI-994, ENT, and PAN induced the strongest effects, i.e., a 3.07-, 3.13-, and 2.87-fold increase, respectively (Figure 2A). In line with this, uptake of [^111^In]In-DOTATATE was significantly enhanced, i.e., 8.14-, 8.30-, and 7.54-fold, respectively (Figure 2B). Surprisingly, even though VPA had a relatively modest effect on the *SSTR2* mRNA expression level, the uptake of [^111^In]In-DOTATATE after VPA was most pronounced, i.e., an 8.63-fold increase in uptake.

In NCI-H727 cells, the observed increases in percentage of internalized and membrane-bound fractions of [^111^In]In-DOTATATE upon HDACi treatment followed similar patterns, demonstrated by a significant positive correlation (r^2^ = 0.9565; *p* = 0.0007) (Figure S2a). Both the membrane and internalized fractions of radioactivity were most strongly increased upon VPA treatment: 4.16- and 4.45-fold, respectively. Internalization of [^111^In]In-DOTATATE was also significantly increased after treatment with CI-994 (1.66-fold), ENT (2.91-fold), LMK-235 (2.10-fold), and PAN (1.58-fold). MOC slightly downregulated the uptake of [^111^In]In-DOTATATE, i.e., 0.32-fold reduced uptake (Figure 2B). *SSTR2* mRNA expression levels followed an identical pattern (Figure 2A), resulting in a significant positive correlation between *SSTR2* mRNA expression levels and [^111^In]In-DOTATATE uptake levels (r^2^ = 0.9005; *p* = 0.0038) (Figure S2B). A statistically significant correlation was not reached in BON-1 cells (r^2^ = 0.4534) (Figure S2C).

### 3.3. Reversibility Profile of SSTR2 Expression in BON-1 and NCI-H727 Cells

**Figure 3:**
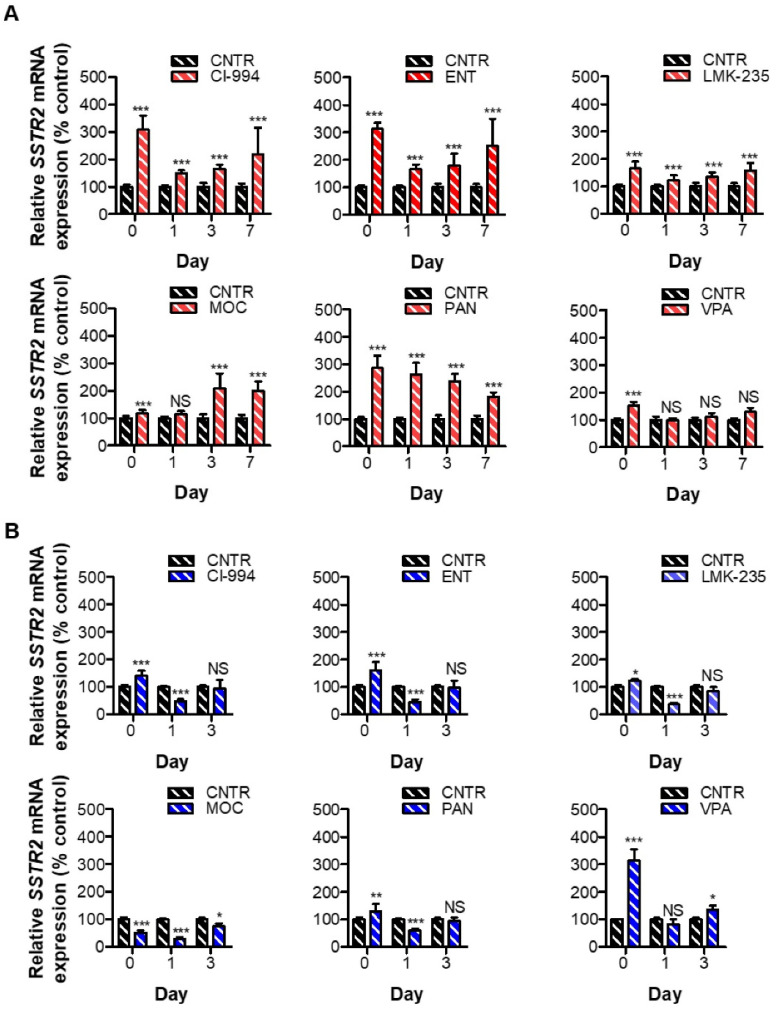
To determine reversibility of SSTR2 upregulation upon HDACi withdrawal, *SSTR2* mRNA levels were determined by RT-qPCR directly after treatment with HDACi at the IC_50_ dose (D0), and one (D1), three (D3), and seven days (D7) after HDACi withdrawal for BON-1 (**A**), and at D0, D1, and D3 for NCI-H727 (**B**). Results were normalized to vehicle-treated cells. * *p* < 0.05, ** *p* < 0.01, *** *p* < 0.001, NS; non-significant.

Reversibility profiles of the effects of HDACis in BON-1 cells showed that control expression levels were not reached after treatment with CI-994, ENT, and LMK-235, i.e., 2.19-, 2.51-, and 1.57-fold increase seven days after drug withdrawal, respectively (Figure 3A). For these inhibitors, strong reductions within the first day after HDACi removal were observed. For PAN-treated cells, control levels were also not reached (1.81-fold increase). In these cells, a gradual downregulation of *SSTR2* expression over time was observed, suggesting a slow wash out of the effect of PAN on these cells. For MOC- and VPA-treated BON-1 cells, control levels were already reached after one day. At day three and day seven, a slight increase in *SSTR2* mRNA expression levels was observed in MOC treated cells, reaching significance for both time points.

In NCI-H727 cells, one day after VPA withdrawal control *SSTR2* mRNA expression levels were already observed (Figure 3B). Surprisingly, after three days, a small but significant upregulation was observed, i.e., 1.37-fold. For all other conditions, another reversibility pattern was observed. *SSTR2* expression levels were significantly reduced already one day after drug withdrawal, i.e., 0.53-, 0.55-, 0.63, 0.71-, and 0.43-fold for CI-994, ENT, LMK-235, MOC, and PAN, respectively. Generally, *SSTR2* mRNA expression levels reached control expression levels three days after drug removal. For MOC treatment, the only HDACi inducing *SSTR2* mRNA downregulation directly after treatment, control levels were not reached within this time frame.

### 3.4. Effects of HDACis in GOT1 Cells

**Figure 4:**
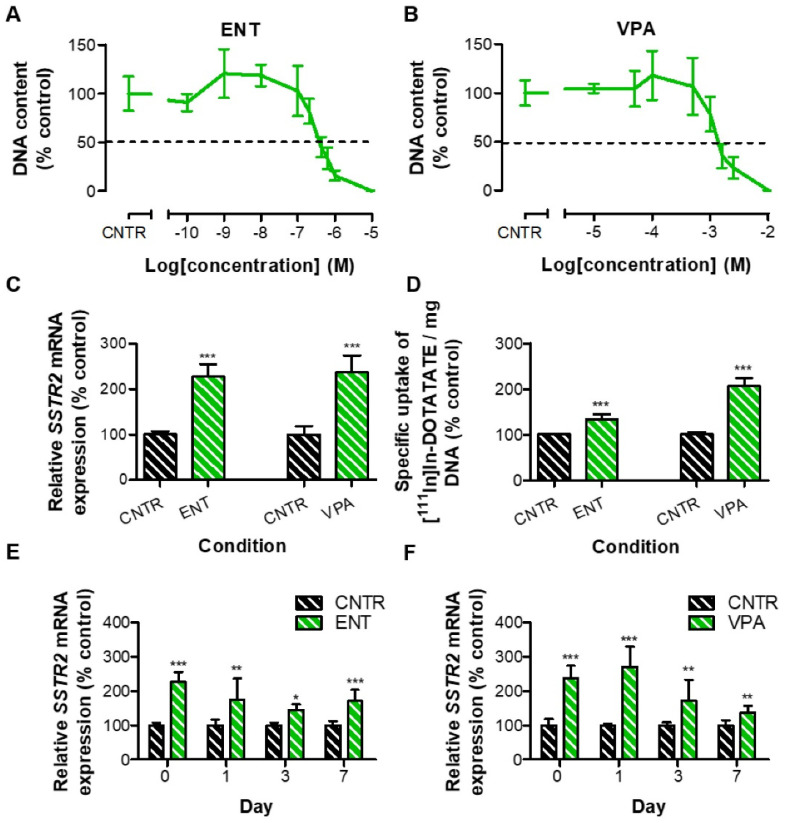
To examine the effect of HDACis in the GOT1 cell line, a dose–response curve was prepared for ENT (**A**) and VPA (**B**). After a seven-day treatment with the IC_50_ dose, the increase in (**C**) *SSTR2* mRNA levels and (**D**) total uptake of radiolabeled SSAs were examined. (**E**,**F**) Reversibility profiles after HDACi withdrawal were evaluated by RT-qPCR directly after treatment (D0), and one (D1), three (D3), and seven days (D7) after HDACi withdrawal. Results in (**C**–**F**) were normalized to vehicle-treated cells. * *p* < 0.05, ** *p* < 0.01, *** *p* < 0.001.

Based on effects induced in BON-1 and NCI-H727 cells, the effects of ENT and VPA were examined in GOT1 cells. Measured IC_50_ values were 384 nM for ENT and 1.36 mM for VPA treatment (Figure 4A,B, Table S2). Both HDACis significantly increased *SSTR2* mRNA expression levels: 2.27- and 2.37-fold, respectively (Figure 4C). In line with this, also the total uptake of [^111^In]In-DOTATATE was significantly enhanced, i.e., 1.34- and 2.06-fold, respectively (Figure 4D).

### 3.5. Reversibility Profile of SSTR2 Expression in GOT1 Cells

Concordant with the observations in BON-1 and NCI-H727 cells, the effects of ENT and VPA gradually decreased over the seven-day period. However, control *SSTR2* mRNA expression levels were not reached within the examined time period (Figure 4D,E).

### 3.6. Radiosensitizing Effects upon VPA Treatment

**Figure 5:**
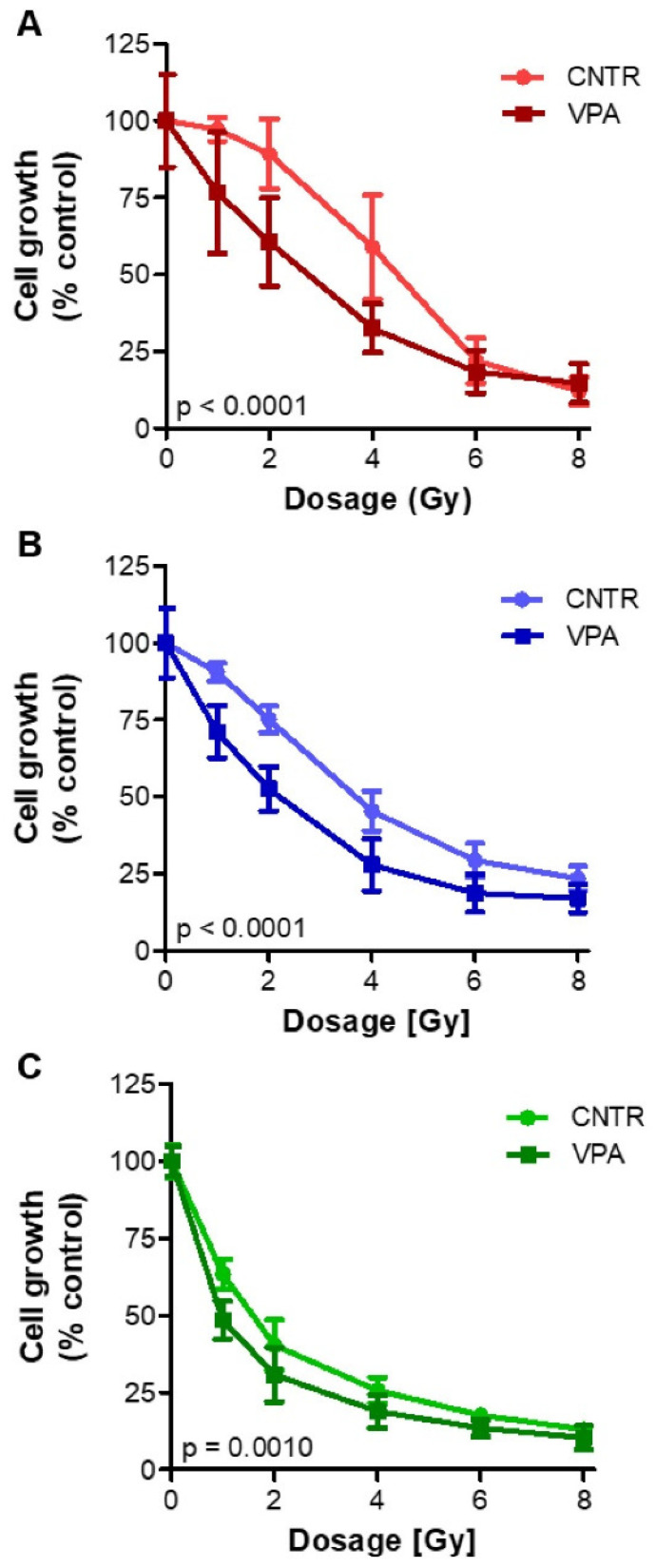
After a seven-day treatment with the IC_50_ dose, VPA-treated and untreated BON-1 (**A**), NCI-H727 (**B**), and GOT1 (**C**) cells were exposed to different dosages of irradiation using external beam radiation to investigate increased radiosensitivity.

As VPA treatment induced strong and significant stimulatory effects on *SSTR2* mRNA expression and [^111^In]In-DOTATATE uptake in all three NET cell lines, the effect of this HDACi was also examined in terms of radiosensitivity (Figure 5). Irradiated VPA-treated NET cells proliferated less than irradiated control cells. IC_50_ values were 4.39 Gy, 3.22 Gy, and 1.34 Gy for untreated BON-1, NCI-H727, and GOT-1 cells, whereas VPA-treated cells had IC_50_ values of 2.82 Gy, 1.88 Gy, and 0.72 Gy, respectively, demonstrating a statistically significant increased sensitivity to external beam irradiation induced by VPA.

## 4. Discussion

As the number of complete responses in NET patients is limited after PRRT, therapy improvement is highly needed. For this reason, we focused on HDACi-induced SSTR2 upregulation in three NET cell-line models as a way of improving uptake of radiolabeled SSAs, and thereby possibly increasing treatment efficacy.

In line with our results, previous studies also reported on the effects of HDACi treatment, showing SSTR2 upregulation and/or increased uptake of radiolabeled SSAs in different NET models [16,19,20,21,22,23,24,25,26]. However, the strength of our study was the use of HDACi-specific and cell line-based IC_50_ values allowing for a comparison of effects induced by HDACis targeting different classes of HDAC enzymes. Moreover, we investigated the induced effects in three frequently used NET cell-line models derived from different origins. Importantly, the established IC_50_ values of the HDACis were below or within the same order of magnitude as the therapeutic dose, indicating the clinical relevance of the used concentrations.

The effects induced by the HDACis clearly enhanced uptake of the radiolabeled SSA [^111^In]In-DOTATATE, as well as *SSTR2* mRNA expression levels in both BON-1 and NCI-H727 cells. VPA and ENT induced the strongest effects in both cell lines. CI-994, targeting the same HDAC enzymes as VPA and ENT, also induced strong upregulation in BON-1 cells, suggesting that epigenetic modifiers targeting HDAC class I enzymes are strongly involved in SSTR2 transcription. Therefore, we expected that MOC, targeting both HDAC class I and class IV, would also enhance SSTR2 expression. However, only weak *SSTR2* upregulation and even downregulation was observed, suggesting that HDACis may have induced cell-specific responses.

The extent of receptor upregulation upon HDACi treatment on *SSTR2* mRNA expression level and uptake of [^111^In]In-DOTATATE correlated significantly in NCI-H727 cells (r^2^ = 0.9005). For BON-1 cells, an r^2^ of 0.4534 was obtained. This correlation was not statistically significant due to the potent effects of VPA treatment on the uptake of [^111^In]In-DOTATATE, i.e., imbalance between mRNA and uptake levels. With the exception of this condition, all other conditions indicated that the enhanced uptake was caused by SSTR2 upregulation, instead of other mechanisms-of-actions, e.g., faster recycling of SSTR2 to the cell membrane after internalization. Next to the correlation between *SSTR2* mRNA expression levels and uptake of [^111^In]In-DOTATATE, it has been demonstrated in literature that the uptake of radiolabeled SSTR2 analogue is associated with SSTR2 protein expression levels [27,28]. We therefore hypothesized that HDACi treatment increased the uptake of [^111^In]In-DOTATATE by upregulation of SSTR2 protein expression levels.

In our study, we also evaluated whether SSTR2 baseline expression levels were associated with the extent of HDACi-induced SSTR2 upregulation. In general, our results showed that the strongest effects were induced in BON-1 cells, e.g., 8.63-, 4.16-, and 2.06-fold increased uptake of [^111^In]In-DOTATATE after VPA treatment in BON-1, NCI-H727, and GOT1 cells, respectively. This pattern was observed for the majority of HDACis, suggesting that there may have been a relationship between extent of receptor upregulation and SSTR2 baseline expression levels. In the study of Exner et al. [29], it was demonstrated that *SSTR2* mRNA expression levels of BON-1 and NCI-H727 cells are lower than or comparable to control pancreatic and ileum tissue, respectively. Since GOT1 has higher *SSTR2* mRNA expression, we hypothesized that these cells had more expression than control tissue. We thereby demonstrated that HDACis could upregulate SSTR2 expression in NET cell lines characterized by a broad range of baseline expression levels, with SSTR2 expression levels lower and higher than control tissue.

To the best of our knowledge, we are the first reporting on the reversibility of SSTR2 upregulation after HDACi withdrawal. As epigenetic histone modifications are part of a dynamic process and the resulting marks are therefore reversible, we hypothesized that SSTR2 expression levels would go back to baseline. In line with this, our results showed that effects induced by HDACi treatment were largely and rapidly reversible. Generally, the largest reductions were observed in the first day. One day after drug withdrawal in NCI-H727 cells, a significant reduction was observed in all conditions, which frequently resulted in *SSTR2* downregulation compared to control cells. We hypothesized that upon drug withdrawal, HDACi enzymes were over-activated, resulting in strong histone deacetylation and thus reduced SSTR2 expression levels. However, over a time course of three days, control expression levels were reached again. This quick reversibility upon HDACi withdrawal can provide guidance for the timing between HDACi administration and [^177^Lu]Lu-DOTATATE injection in preclinical studies in order to obtain beneficial effects.

Based on our analysis, VPA induced the strongest effects on uptake of [^111^In]In-DOTATATE in all three examined NET cell lines. Therefore, the effect of VPA on radiosensitivity was examined using external beam irradiation. Using external beam irradiation, the radiosensitizing effect was distinguished from other possible mechanisms, e.g., increased therapeutic effect as a consequence of increased [^177^Lu]Lu-DOTATATE uptake or a combination of these two. We observed that the radiosensitivity of all NET cell lines was significantly increased after VPA treatment. This is in line with earlier published data by Jin et al. [19] who showed a slightly increased radiosensitivity upon treatment of BON-1 and QGP-1 cells with CI-994. Similar results were demonstrated for several other cancer types [30,31,32,33]. This suggests that VPA may have a dual function; it increases both *SSTR2* mRNA expression levels and uptake of [^111^In]In-DOTATATE, and it increases radiosensitivity towards PRRT. Another major advantage is that VPA is already used in a clinical setting, e.g., for treatment of epilepsy and psychiatric disorders [34,35]. This HDACi can therefore be of great interest for its potency to upregulate SSTR2.

Although upregulation of the target receptor has already been demonstrated to be successful for improving radioligand therapy for NETs in vitro [36] and increasing uptake of radiolabeled somatostatin analogues in vivo [20,22], the improved therapeutic effect on tumor growth in vivo is not established yet for upregulation of the target receptor in combination with [^177^Lu]Lu-DOTATATE treatment. In prostate cancer, several approaches are under investigation to increase target expression levels, i.e., treatment with antiandrogen MDV3100 resulting in an increased uptake of radiolabeled PSMA-targeting antibody ^64^Cu-J591 [37], and, more importantly, treatment with enzalutamide enhancing PSMA expression and thereby improving survival in xenograft models upon combination with PSMA antibody drug conjugates [38]. In contrast to the above-described study by DiPippo et al. [38], Lückerath et al. [39] did not show an increased therapeutic response after treatment with [^177^Lu]Lu-PSMA617 in combination with enzalutamide which caused an increased PSMA expression. In the study of McDevitt et al. [40] a feed-forward loop was described in prostate cancer xenografts irradiated with [^225^Ac]hu11B6, causing upregulation of the human kallikrein peptidase 2, which is targeted by hu11B6 itself. Therefore, the potential of a combinational therapy consisting of HDACi and [^177^Lu]Lu-DOTATATE in NET models should be addressed in future preclinical studies. Moreover, in vivo studies are required to examine the effect of HDACi treatment on SSTR2 expression level itself. There are several variables which should be taken into account, e.g., HDACi dose, treatment duration, and frequency and route of administration. Moreover, the quick reversibility after HDACi withdrawal in vitro, as was observed in our study, indicates that timing between HDACi and DOTATATE injection may be an important factor in preclinical studies. With these in vivo studies, it is also important to determine tumor-to-background ratios, thereby taking into account the effect of HDACis on physiological tissues.

## 5. Conclusions

In conclusion, we demonstrated that SSTR2 upregulation by HDACi treatment was possible in NET cell lines of different origins, especially using HDACi specifically targeting class I HDACs, and with strongest effects observed in cells characterized by low SSTR2 baseline expression levels. Generally, the effects were rapidly and largely reversible within one day after HDACi withdrawal. This suggests that proper timing of HDACi treatment could be an important factor in both preclinical and clinical settings. Future studies will provide definite answers about the potential for this combinational therapy in order to improve NET patient outcomes.

## Data Availability

Data can be requested by contacting the corresponding author.

## Supplementary Materials

The following are available online at https://www.mdpi.com/article/10.3390/cancers13194905/s1, Figure S1: Pearson correlation analysis of *SSTR2* mRNA and [^111^In]In-DOTATATE uptake, Figure S2: Pearson correlation analysis of HDACi-treated cell, Table S1: Primers used for RT-qPCR, Table S2: HDACi IC_50_ values in BON-1, NCI-H727, and GOT1 cells.

## References

[1] Raphael M.J., Chan D.L., Law C., Singh S. (2017). Principles of diagnosis and management of neuroendocrine tumours. Can. Med. Assoc. J..

[2] Rinke A., Müller H.-H., Schade-Brittinger C., Klose K.-J., Barth P., Wied M., Mayer C., Aminossadati B., Pape U.-F., Bläker M. (2009). Placebo-Controlled, Double-Blind, Prospective, Randomized Study on the Effect of Octreotide LAR in the Control of Tumor Growth in Patients With Metastatic Neuroendocrine Midgut Tumors: A Report From the PROMID Study Group. J. Clin. Oncol..

[3] Caplin M.E., Pavel M., Ćwikła J.B., Phan A.T., Raderer M., Sedláčková E., Cadiot G., Wolin E.M., Capdevila J., Wall L. (2014). Lanreotide in Metastatic Enteropancreatic Neuroendocrine Tumors. N. Engl. J. Med..

[4] Strosberg J., El-Haddad G., Wolin E., Hendifar A., Yao J., Chasen B., Mittra E., Kunz P.L., Kulke M.H., Jacene H. (2017). Phase 3 Trial of ^177^Lu-Dotatate for Midgut Neuroendocrine Tumors. N. Engl. J. Med..

[5] Brabander T., Van Der Zwan W.A., Teunissen J.J., Kam B.L., Feelders R.A., De Herder W.W., Van Eijck C.H., Franssen G.J., Krenning E.P., Kwekkeboom D.J. (2017). Long-Term Efficacy, Survival, and Safety of [177Lu-DOTA0,Tyr3]octreotate in Patients with Gastroenteropancreatic and Bronchial Neuroendocrine Tumors. Clin. Cancer Res..

[6] Okuwaki K., Kida M., Mikami T., Yamauchi H., Imaizumi H., Miyazawa S., Iwai T., Takezawa M., Saegusa M., Watanabe M. (2013). Clinicopathologic characteristics of pancreatic neuroendocrine tumors and relation of somatostatin receptor type 2A to outcomes. Cancer.

[7] Klieser E., Urbas R., Stättner S., Primavesi F., Jäger T., Dinnewitzer A., Mayr C., Kiesslich T., Holzmann K., Di Fazio P. (2017). Comprehensive immunohistochemical analysis of histone deacetylases in pancreatic neuroendocrine tumors: HDAC5 as a predictor of poor clinical outcome. Hum. Pathol..

[8] Alvarez M.J., Subramaniam P.S., Tang L.H., Grunn A., Aburi M., Rieckhof G., Komissarova E.V., Hagan E.A., Bodei L., Clemons P.A. (2018). A precision oncology approach to the pharmacological targeting of mechanistic dependencies in neuroendocrine tumors. Nat. Genet..

[9] Di Domenico A., Wiedmer T., Marinoni I., Perren A. (2017). Genetic and epigenetic drivers of neuroendocrine tumours (NET). Endocr.-Relat. Cancer.

[10] Klomp M., Dalm S., de Jong M., Feelders R., Hofland J. (2021). Epigenetic regulation of somatostatin and somatostatin receptors in neuroendocrine tumors and other types of cancer. Rev. Endocr. Metab. Disord..

[11] Alhamwe B.A., Khalaila R., Wolf J., von Bülow V., Harb H., Alhamdan F., Hii C.S., Prescott S.L., Ferrante A., Renz H. (2018). Histone modifications and their role in epigenetics of atopy and allergic diseases. Allergy Asthma Clin. Immunol..

[12] Parbin S., Kar S., Shilpi A., Sengupta D., Deb M., Rath S.K., Patra S.K. (2014). Histone deacetylases: A saga of perturbed acetylation homeostasis in cancer. J. Histochem. Cytochem..

[13] Hadden M.J., Advani A. (2018). Histone Deacetylase Inhibitors and Diabetic Kidney Disease. Int. J. Mol. Sci..

[14] Hessmann E., Johnsen S., Siveke J.T., Ellenrieder V. (2016). Epigenetic treatment of pancreatic cancer: Is there a therapeutic perspective on the horizon?. Gut.

[15] Marek L., Hamacher A., Hansen F.K., Kuna K., Gohlke H., Kassack M.U., Kurz T. (2013). Histone Deacetylase (HDAC) Inhibitors with a Novel Connecting Unit Linker Region Reveal a Selectivity Profile for HDAC4 and HDAC5 with Improved Activity against Chemoresistant Cancer Cells. J. Med. Chem..

[16] Veenstra M.J., Van Koetsveld P.M., Dogan F., Farrell W.E., Feelders R.A., Lamberts S.W., De Herder W.W., Vitale G., Hofland L.J. (2016). Epidrug-induced upregulation of functional somatostatin type 2 receptors in human pancreatic neuroendocrine tumor cells. Oncotarget.

[17] de Blois E., Chan H.S., de Zanger R., Konijnenberg M., Breeman W.A. (2014). Application of single-vial ready-for-use formulation of 111In- or 177Lu-labelled somatostatin analogs. Appl. Radiat. Isot..

[18] Dalm S.U., Nonnekens J., Doeswijk G.N., de Blois E., van Gent D.C., Konijnenberg M.W., de Jong M. (2016). Comparison of the Therapeutic Response to Treatment with a 177Lu-Labeled Somatostatin Receptor Agonist and Antagonist in Preclinical Models. J. Nucl. Med..

[19] Jin X.-F., Auernhammer C.J., Ilhan H., Lindner S., Nölting S., Maurer J., Spoettl G., Orth M., Spöttl G. (2019). Combination of 5-Fluorouracil with Epigenetic Modifiers Induces Radiosensitization, Somatostatin Receptor 2 Expression, and Radioligand Binding in Neuroendocrine Tumor Cells In Vitro. J. Nucl. Med..

[20] Taelman V.F., Radojewski P., Marincek N., Ben-Shlomo A., Grotzky A., Olariu C.I., Perren A., Stettler C., Krause T., Meier L.P. (2016). Upregulation of Key Molecules for Targeted Imaging and Therapy. J. Nucl. Med..

[21] Wanek J., Gaisberger M., Beyreis M., Mayr C., Helm K., Primavesi F., Jäger T., Di Fazio P., Jakab M., Wagner A. (2018). Pharmacological Inhibition of Class IIA HDACs by LMK-235 in Pancreatic Neuroendocrine Tumor Cells. Int. J. Mol. Sci..

[22] Guenter R., Aweda T., Matos D.M.C., Jang S., Whitt J., Cheng Y.-Q., Liu X.M., Chen H., Lapi S.E., Jaskula-Sztul R. (2020). Overexpression of somatostatin receptor type 2 in neuroendocrine tumors for improved Ga68-DOTATATE imaging and treatment. Surgery.

[23] Guenter R.E., Aweda T., Matos D.M.C., Whitt J., Chang A.W., Cheng E.Y., Liu X.M., Chen H., Lapi S.E., Jaskula-Sztul R. (2019). Pulmonary Carcinoid Surface Receptor Modulation Using Histone Deacetylase Inhibitors. Cancers.

[24] Torrisani J., Hanoun N., Laurell H., Lopez F., Maoret J.-J., Souque A., Susini C., Cordelier P., Buscail L. (2008). Identification of an Upstream Promoter of the Human Somatostatin Receptor, hSSTR2, Which Is Controlled by Epigenetic Modifications. Endocrinology.

[25] Arvidsson Y., Johanson V., Pfragner R., Wängberg B., Nilsson O. (2015). Cytotoxic Effects of Valproic Acid on Neuroendocrine Tumour Cells. Neuroendocrinology.

[26] Sun L., Qian Q., Sun G., Mackey L.V., Fuselier J.A., Coy D.H., Yu C.-Y. (2016). Valproic acid induces NET cell growth arrest and enhances tumor suppression of the receptor-targeted peptide–drug conjugate via activating somatostatin receptor type II. J. Drug Target..

[27] Miederer M., Seidl S., Buck A., Scheidhauer K., Wester H.-J., Schwaiger M., Perren A. (2008). Correlation of immunohistopathological expression of somatostatin receptor 2 with standardised uptake values in 68Ga-DOTATOC PET/CT. Eur. J. Nucl. Med. Mol. Imaging.

[28] Yu J., Cao F., Zhao X., Xie Q., Lu M., Li J., Yang Z., Sun Y. (2021). Correlation and Comparison of Somatostatin Receptor Type 2 Immunohistochemical Scoring Systems with ^68^Ga-DOTATATE Positron Emission Tomography/Computed Tomography Imaging in Gastroenteropancreatic Neuroendocrine Neoplasms. Neuroendocrinology.

[29] Exner S., Prasad V., Wiedenmann B., Grötzinger C. (2018). Octreotide Does Not Inhibit Proliferation in Five Neuroendocrine Tumor Cell Lines. Front. Endocrinol..

[30] Kubota N., Kuribayashi T., Ohara M., Sora S. (2009). Scriptaid, a novel histone deacetylase inhibitor, enhances the response of human tumor cells to radiation. Int. J. Mol. Med..

[31] Li H., Ma L., Bian X., Lv Y., Lin W. (2021). FK228 sensitizes radioresistant small cell lung cancer cells to radiation. Clin. Epigenet..

[32] Chen X., Wong P., Radany E., Wong J.Y. (2009). HDAC Inhibitor, Valproic Acid, Induces p53-Dependent Radiosensitization of Colon Cancer Cells. Cancer Biotherapy Radiopharm..

[33] Munshi A., Kurland J.F., Nishikawa T., Tanaka T., Hobbs M.L., Tucker S.L., Ismail S., Stevens C., Meyn R.E. (2005). Histone Deacetylase Inhibitors Radiosensitize Human Melanoma Cells by Suppressing DNA Repair Activity. Clin. Cancer Res..

[34] Nanau R.M., Neuman M.G. (2013). Adverse drug reactions induced by valproic acid. Clin. Biochem..

[35] Romoli M., Mazzocchetti P., D’Alonzo R., Siliquini S., Rinaldi V.E., Verrotti A., Calabresi P., Costa C. (2019). Valproic Acid and Epilepsy: From Molecular Mechanisms to Clinical Evidences. Curr. Neuropharmacol..

[36] Shah R.G., Merlin M.A., Adant S., Zine-Eddine F., Beauregard J.-M., Shah G.M. (2021). Chemotherapy-Induced Upregulation of Somatostatin Receptor-2 Increases the Uptake and Efficacy of ^177^Lu-DOTA-Octreotate in Neuroendocrine Tumor Cells. Cancers.

[37] Evans M.J., Smith-Jones P.M., Wongvipat J., Navarro V., Kim S., Bander N.H., Larson S.M., Sawyers C.L. (2011). Noninvasive measurement of androgen receptor signaling with a positron-emitting radiopharmaceutical that targets prostate-specific membrane antigen. Proc. Natl. Acad. Sci. USA.

[38] DiPippo V.A., Nguyen H.M., Brown L.G., Olson W.C., Vessella R.L., Corey E. (2016). Addition of PSMA ADC to enzalutamide therapy significantly improves survival in in vivo model of castration resistant prostate cancer. Prostate.

[39] Lückerath K., Wei L., Fendler W.P., Axelsson S.E., Stuparu A.D., Slavik R., Mona C.E., Calais J., Rettig M., Reiter R.E. (2018). Preclinical evaluation of PSMA expression in response to androgen receptor blockade for theranostics in prostate cancer. EJNMMI Res..

[40] McDevitt M.R., Thorek D.L.J., Hashimoto T., Gondo T., Veach D.R., Sharma S.K., Kalidindi T.M., Abou D.S., Watson P.A., Beattie B.J. (2018). Feed-forward alpha particle radiotherapy ablates androgen receptor-addicted prostate cancer. Nat. Commun..

