# Supplementary material for: Comparing the Effect of Multiple Histone Deacetylase Inhibitors on SSTR2 Expression and [111In]In-DOTATATE Uptake in NET Cells
Source: Cancers (Basel). 2021 Sep 29;13(19):4905. doi: 10.3390/cancers13194905 (PMC8508045; doi:10.3390/cancers13194905)
Supplement: Supplementary file 1 [file cancers-13-04905-s001.zip › cancers-1366105-supplementary.pdf]

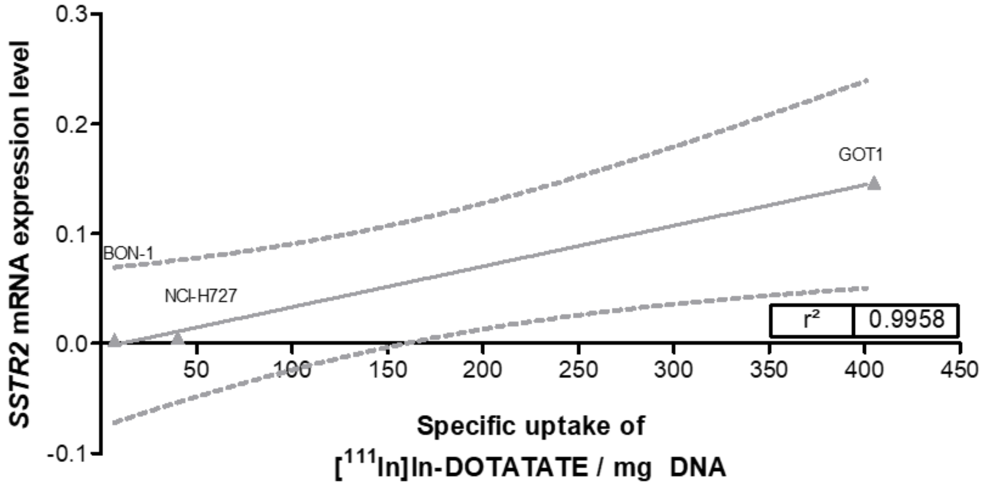


**FIGURE S1.** Pearson correlation analysis was performed by plotting basal *SSTR2* mRNA expression level in BON-1, NCI-H727 and GOT1 cells against corresponding uptake of [$^{111}$In]In-DOTATATE per milligram of DNA.

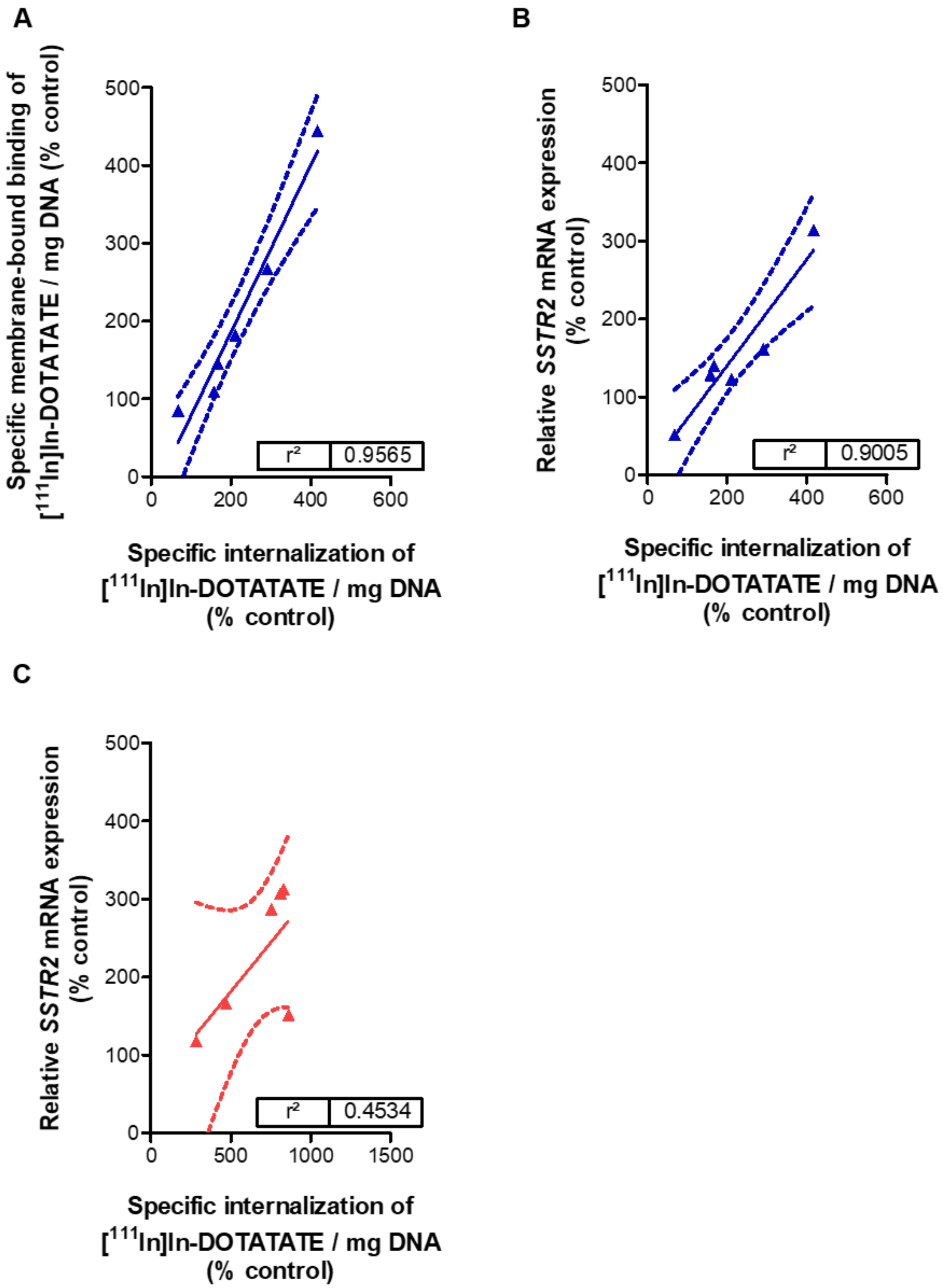
A
Specific membrane-bound binding of
[111In]In-DOTATATE / mg DNA (% control)
500
400
300
200
100
0
0
200
400
600
r²
0.9565
Specific internalization of
[111In]In-DOTATATE / mg DNA
(% control)
B
Relative SSTR2 mRNA expression
(% control)
500
400
300
200
100
0
0
200
400
600
r²
0.9005
Specific internalization of
[111In]In-DOTATATE / mg DNA
(% control)
C
Relative SSTR2 mRNA expression
(% control)
500
400
300
200
100
0
0
500
1000
1500
r²
0.4534
Specific internalization of
[111In]In-DOTATATE / mg DNA
(% control)

**FIGURE S2.** Pearson correlation analysis was performed by plotting the HDACi-enhanced [$^{111}$In]In-DOTATATE membrane-bound fraction to the increased internalized fraction in NCI-H727 cells (A), and by plotting the HDACi-enhanced [$^{111}$In]In-DOTATATE internalization levels against increased *SSTR2* mRNA expression levels in BON-1 (B) and NCI-H727 (C) cells.

| Gene | Primer information | Efficacy factor |
|---|---|---|
| **GUSB** | Hs00939627_m1 | 1.95 |
| **HPRT1** | Hs02800695_m1 | 1.97 |
| **β-Actin** | Hs01060665_g1 | 1.96 |
| **SSTR2** | Forward: 5'-TCG GCC AAG TGG AGG AGA C<br>Reverse: 3'-AGA GAC TCC CCA CAC AGC CA<br>Probe: CCGGACGGCCAAGATGATCACC | 1.91 |

**TABLE S1.** Primers used for RT-qPCR. HKG primers (Thermo Fisher Scientific) were diluted twenty times. For SSTR2, final concentration were 0.5 pM for both forward and reverse primers, and 0.1 pM for the SSTR2 probe (Sigma).

| HDACi | BON-1 | NCI-H727 | GOT1 |
|---|---|---|---|
| **CI-994** | 1.85 µM | 3.05 µM | ND |
| **ENT** | 218 nM | 315 nM | 384 nM |
| **LMK235** | 154 nM | 348 nM | ND |
| **MOC** | 84.3 nM | 171 nM | ND |
| **PAN** | 3.11 nM | 8.53 nM | ND |
| **VPA** | 1.12 mM | 1.31 mM | 1.36 mM |

**TABLE S2.** $IC_{50}$ values on growth of BON-1, NCI-H727 and GOT1 cell lines, based on a seven day HDACi treatment period.
